# Balancing the duty to treat with the duty to family in the context of the COVID-19 pandemic

**DOI:** 10.1136/medethics-2020-106250

**Published:** 2020-04-24

**Authors:** Doug McConnell

**Affiliations:** Philosophy, University of Oxford, Oxford 2678, UK

**Keywords:** public health ethics, professional - professional relationship, family, health workforce

## Abstract

Healthcare systems around the world are struggling to maintain a sufficient workforce to provide adequate care during the COVID-19 pandemic. Staffing problems have been exacerbated by healthcare workers (HCWs) refusing to work out of concern for their families. I sketch a deontological framework for assessing when it is morally permissible for HCWs to abstain from work to protect their families from infection and when it is a dereliction of duty to patients. I argue that it is morally permissible for HCWs to abstain from work when their duty to treat is outweighed by the combined risks and burdens of that work. For HCWs who live with their families, the obligation to protect one’s family from infection contributes significantly to those burdens. There are, however, a range of complicating factors including the strength of duty to treat which varies according to the HCW’s role, the vulnerability of family members to the disease, the willingness of family members to risk infection and the resources available to the HCW to protect their family. In many cases, HCWs in ‘frontline’ roles with a weak duty to treat and families at home will be morally permitted to abstain from work given the risks posed by COVID-19; therefore, society should provide additional incentives to maintain sufficient staff in these roles.

## Introduction

The COVID-19 pandemic is overwhelming the healthcare systems of several countries and, in some cases, drastically reducing the standard of care that can be provided.^1^ This is not only due to insufficient physical resources, such as ventilators, but insufficient healthcare workers (HCWs).^1^ The staffing problem is exacerbated by the fact that, at any one time, a significant proportion of HCWs will themselves be infected and unable to work due to being ill and contagious.^2^ A lack of staff increases patient mortality because early warning signs are missed and treatment is provided late, if at all. Furthermore, the staff that *are* available become fatigued and make more mistakes, some of which increase the transmission of infectious disease. Therefore, maintaining a sufficient workforce is a high priority for healthcare systems during an epidemic.

An important factor in staff availability is whether HCWs are *willing* to work. In a survey of employees at a university hospital, 24% of physicians and 26% of nurses thought that it was ethical to abstain from work to protect themselves or their families during a pandemic.^3^ Another survey found that concern for family safety was the most frequently cited factor in reducing willingness to work during a pandemic.^4^ So it isn’t unsurprising that there have been cases of HCWs refusing to work in order to protect their families during the COVID-19 pandemic. Social care workers at an aged-care home in Australia refused to work after a COVID-19 outbreak at their facility, citing concern for their family members, some of whom were immunocompromised.^5^ Australian disability service organisations have reported ‘enormous trouble with workers not turning up’.^6^ Physicians and nurses in the UK have threatened to quit because a lack of adequate personal protective equipment (PPE) is putting them and their families at too much risk.^7 8^ Most shockingly, elderly patients in Spain appear to have been abandoned in their aged-care facility with some being found dead in their beds.^9^

These cases raise the following questions. When is it morally permissible for HCWs to abstain from work to protect their families from infectious disease and when is it a dereliction of duty to their patients? When can society pressure HCWs to meet a moral obligation to work during an epidemic and when would such pressure be unjustified? I set out a deontological framework for answering these questions and apply it in the context of the COVID-19 pandemic.

The most important factor counting in favour of an obligation to work is the HCW’s duty to treat, the strength of which depends on the HCW’s role. The duty to treat can be outweighed by various risks and burdens, including the burden of protecting one’s family from infection. There are several factors that adjust the burden of protecting one’s family including the protective resources available to the HCW, the family members’ vulnerability to the disease and the family members’ willingness to risk infection.

### COVID-19 risks and burdens

Before we can say what HCW’s obligations are, we need an appreciation of the risks and burdens involved in working during the COVID-19 pandemic. I will assume that HCWs are properly informed of these risks and burdens to sidestep the complications that arise in cases of uncertainty and misbelief.

Regarding burdens, HCWs will be asked to work longer shifts with fewer days off in a more stressful environment while wearing uncomfortable PPE much of the time. The fatality rate of HCWs who contract COVID-19 is about 0.6% or ~1/200^10^ with 15% of infections being ‘serious’ or ‘severe’.^11^ Roughly 20% of ‘frontline’ HCWs could expect to become infected at their place of work,^2^ which means that around 0.12% (ie, 0.6% of 20%) or ~1/1000 of ‘frontline’ HCWs would be expected to die of COVID-19. Presumably those in roles with less exposure to COVID-19 face less risk. Those are the risks and burdens for HCWs regardless of whether they have families at home. For HCWs who have families at home, there are further burdens.

The fatality rate for those under 70 years of age and in good health are roughly the same as for HCWs. COVID-19 is a much worse proposition, however, for the elderly and those with comorbid cardiovascular disease, diabetes, chronic respiratory disease, hypertension or cancer.^11^ The fatality rate is 8% for those aged 70 to 79 years and nearly 15% for those aged 80 years and above.^11^ Many HCWs will have family members in these vulnerable groups. As a rough indication of the risk to family members, consider a ‘frontline’ nurse who shares a home with his 80-year-old father. There could be as much as a 3% chance of passing on a fatal infection (ie, a 20% chance of the HCW being infected at work and a 15% chance infection is fatal in the father’s age group). This risk to family can be decreased if the HCW takes careful measures not to pass on the infection in the home but, given that people are infectious prior to exhibiting symptoms, only highly burdensome measures will be effective. A ‘frontline’ nurse in Ireland details some of the effort involved:

I’ll come home from work through my laundry room door that leads to the outside. I’ll strip naked including shoes and put everything straight into the washing machine on sanitise mode. I’ll use a Clorox wipe to clean anything I touched in the process. I’ll then take the towel that my husband has left for me and use it to walk to my master bedroom covered up. In there, a room that nobody else is allowed to enter after today… after my shower I’ll sanitise everything I touched again, then hand sanitise and get dressed. When I’m done with this process I’ll be able to sit in the family room 6 feet away from everyone I love.^12^

These measures are likely to be required for weeks if not months and, even then, they might not prevent the HCW infecting their family. Furthermore, many HCWs living arrangements won’t allow for such effective segregation and they will be unable to reduce the risk to family as effectively. So, why would HCWs be obliged to take on any of these risks and burdens?

### Minimally decent Samaritanism

As a starting point, it’s widely agreed that there is a duty to be a minimally decent Samaritan, that is, one should go out of one’s way to help another if it entails little cost to oneself.^13^ There is a moral obligation to try and save a child drowning in a pond despite the damage it will do to one’s shoes, for example. But one is not obliged to attempt a rescue that would involve a serious risk to one’s own life. Somewhere between the easy and the dangerous rescue there is a threshold where the risks become sufficiently high that one is no longer morally obliged to attempt the rescue.

Like anyone, HCWs are morally obliged to be minimally decent Samaritans. Do the moral demands of minimally decent Samaritanism require HCWs take on the risks and burdens associated with treating COVID-19 to save several (perhaps many) lives? I assume not; not least because it would require the Samaritan to put his/her life on hold for months.^2^ But, of course, the question of whether HCWs should work during the COVID-19 pandemic is not exactly like the question of whether one should rescue several strangers. The patients in need of help have been admitted to government regulated healthcare facilities and the HCWs are employed at those facilities to care for those patients. This situation means that HCWs have a more demanding duty than minimally decent Samaritanism, namely, a duty to treat.

### Duty to treat

It is widely believed that HCWs have a duty to treat, which requires taking on more risk and burden to treat patients than would be expected of a minimally decent Samaritan (even one who somehow happened to have the necessary skills).^14 15^

One justification for the view that HCWs have a duty to treat is that they consent to it when (autonomously) accepting the role.^16^ Of course, the exact risks and burdens one should take to fulfil the duty to treat are not stated explicitly in one’s work contract; they depend on society’s expectations of the role and the HCW is thought to consent to them implicitly. For risk to be implicitly consented to, it must be inherent to the role. A physician specialised in the treatment of infectious disease, for example, could reasonably be thought to have implicitly consented to more risk of infection but not something unrelated like risk of assault. This means that each HCW’s duty to treat involves different risks and burdens depending on the characteristics of their role. However, since all HCW’s roles inherently involve the delivery of good care, there is an obligation to deliver that care in a range of adverse circumstances, including circumstances that involve some risk of infection and some burden to protect one’s family from infection. Those HCW roles that inherently involve treating infectious disease oblige workers in those roles to take some *further* risk of infection and to bear a *greater* burden to protect their families from infection than HCWs not in such roles.

Another justification for the duty to treat appeals to reciprocity between HCWs and society.^16^ HCWs have had their training and knowledge subsidised by the public in various ways and, through licensure, society ensures that some healthcare roles have reduced competition, higher incomes and greater social prestige. In exchange for these benefits, workers have a duty to treat despite higher risks. The reciprocity argument entails that the strength of the duty to treat is proportional to those benefits. Specialised physicians receive substantial benefits so have a very strong duty to treat. Towards the other end of the scale, social care workers receive substantially less from society than physicians and nurses, so their duty to treat is much weaker. Ideally, the role expectations that HCWs implicitly consent to would be justified in light of the benefits society provides and we can see that this is roughly true—society expects more of physicians than nurses, and much more of both physicians and nurses than social care workers.^3^

This analysis of the duty to treat allows us to make relational claims such as: HCWs are obliged to take on more risk and burden to care for patients than minimally decent Samaritans are expected to take to help strangers; physicians are obliged to take on more risk and burden to care for patients than social care workers; specialists in treating infectious diseases are obliged to take more risk of infection than non-specialists. But it is difficult to say exactly how much a duty to treat demands of HCWs in *absolute* terms just as it is difficult to say exactly how much minimally decent Samaritanism demands of us to save the life of a stranger.

Without adding the burden of protecting family to the equation, I estimate that even social care workers’ weak duty to treat would be sufficient to oblige them to take on the personal risk and burden of working through the COVID-19 pandemic. This is based on the assumptions that they will prevent several patients from dying while the personal costs will be long stressful shifts and no more than ~1/1000 chance of fatal infection. If this is right, then all HCWs with stronger duties to treat would also be morally obliged to take on those risks and burdens.

HCWs who lack access to adequate PPE, however, face greater personal risks. Frontline workers with no prospect of PPE, for example, would almost certainly become infected and thus face a ~1/200 chance of death. Even a strong duty to treat, in my estimate, would be insufficient to oblige someone to work with that risk. If this is right, then those frontline HCWs in the UK threatening to quit over a lack of adequate PPE are justified in their complaint but should be permitted to abstain without losing their jobs.^7 8^ HCWs who happen to be more vulnerable to COVID-19 also face higher risks which might excuse them from treating patients on the ‘frontline’, especially those HCWs with a weaker duty to treat.

In the final section, I explore how the additional burden created by a duty to protect one’s family entails that the duty to treat is overridden in a wider range of cases.

### Managing conflict between duty to treat and duty to family

It is morally wrong to negligently harm others, including by negligently infecting them with a harmful disease.^14 17^ During a pandemic, HCWs are more likely to carry the infectious disease causing the pandemic so they ought to take more burdensome measures than usual to avoid negligently infecting others. The measures that one is obliged to take depend on one’s relationship with the other and the risk one will infect them. HCWs have a fiduciary relationship with their patients which entails an obligation to work harder to prevent negligent harm to their patients than to strangers. However, HCW’s obligation to prevent negligent harm to people they are in close relationships with, such as immediate family, is even stronger.^4^ 18 This is because the deep mutual trust and love that characterise these relationships entail proportionally strong obligations, including an obligation to avoid negligently harming each other. The obligations involved in close relationships can also be asymmetrical, such as when people voluntarily enter into caring relationships with their children or elderly parents.

People in such relationships with decision-making capacity can, of course, autonomously waive some of their usual claim to not being harmed. In a pandemic, they might do so to reduce the burden on HCWs in their family. To the extent family members waive their claim to not being harmed by infection, the question of whether the HCW is obliged to work depends solely on the personal costs to the HCW. In what follows I assume family members cannot or have not waived this claim.

Outside of epidemics, HCWs typically manage to fulfil both their duty to treat and their duty to protect their family members from infection. The conditions created by an epidemic, however, make this unusually challenging and, in some circumstances, the burden of protecting one’s family overrides the duty to treat. To illustrate, let’s assume that the measures taken by the Irish nurse above—separating a room in the home for her exclusive use and keeping six feet away from her family at all times—are required for any ‘frontline’ HCW to meet their obligation to close family at home. Does the strength of the HCW’s duty to treat oblige her to take on that burden along with the other personal risks and burdens detailed above? My intuition is that it does for those with a strong duty to treat, such as physicians and nurses. Although I expect that some won’t share this intuition and this case might fall into the large grey area created by uncertainty over the exact strength of the duty to treat. I think it is less controversial, however, to claim that HCWs with a weak duty to treat are not obliged to take on such a burden to protect their families. Social care workers, for example, can plausibly argue that, since society doesn’t provide them with much in the way of benefits, they are not obliged to take such burdensome measures. If that is correct, then the absentee Australian social care workers were morally permitted to abstain from work after the outbreak of COVID-19 in their workplace.^5^ This is even more clearly the case for those two workers who reported having to protect immunocompromised family members and so would have to take on an even greater burden to protect them. This line of argument does not, however, justify abandoning people in aged-care homes, as appears to have happened in Spain.^9^ Even in cases where social care workers are morally permitted to abstain from work to protect family, they are still obliged to inform management so that alternative measures can be taken.

Of course, if HCWs with a weak duty to treat and a family at home are morally permitted to abstain from work, this will exacerbate staffing shortages and patients might still be left without treatment. One solution to this problem is for society to temporarily employ HCWs in roles with a stronger duty to treat. The risks involved in these roles would be more explicit and so more clearly consented to, and the roles would come with clear benefits such as greater pay, prioritised healthcare for HCW’s and their families or accommodation and board for HCWs.

Finally, some HCWs will be caught in circumstances where they will inevitably fail in either their duty to treat or their duty to family. Consider, for example, a ‘frontline’ HCW with a strong duty to treat but no available means of sufficiently reducing the risk of infecting his family (perhaps he lives in a small house with a large family and no alternative living arrangements are available). In cases where the family members are not particularly vulnerable and so are unlikely to be severely harmed, the duty to treat trumps the duty to family and the HCW is morally obliged to work. Conversely, for a HCW in the same situation but with an extremely vulnerable family member at home, the duty to family trumps the duty to treat and the HCW is permitted to abstain from work. But in both kinds of case, compensation is owed for the duty violated. In the first kind of case, public healthcare has benefitted at the expense of the HCW’s family so fairness requires society compensate the family, perhaps with prioritised healthcare. In the second kind of case, the HCW has been relieved of the excessive burden of protecting a vulnerable family member at the expense of public healthcare, so fairness requires the HCW compensate society, perhaps financially or with free labour after the epidemic.

During an epidemic, it is morally permissible for HCWs to abstain from work when their duty to treat is outweighed by the combined risks and burdens of that work. For HCWs who live with their families, the obligation to protect one’s family from infection contributes significantly to those burdens. This is especially the case with COVID-19 which poses significantly more risk to elderly family members than HCWs themselves. In many cases, HCWs in ‘frontline’ roles with a weak duty to treat and families at home will be morally permitted to abstain from work, so society should provide extra incentives to ensure these workers are fairly compensated and to prevent staff shortages in these areas.

## Footnotes

I use the inclusive term ‘healthcare workers’ to refer to whoever provides care to patients, including physicians, nurses, paramedics, social care workers and radiologists among others.

If one thinks that this is required of minimally decent Samaritans then it will follow that HCWs of all kinds are obliged to work in nearly all the circumstances that arise in the treatment of COVID-19 (in developed countries at least).

Of course, society’s expectations and the benefits it provides to HCWs can come apart and then the strength of the duty to treat becomes less clear; is it determined by what was implicitly consented to, the benefits society provides or some fusion of the two? Due to space constraints I won’t pursue this issue here.

This category of close relationships includes relationships with non-family members but for simplicity I limit my discussion to the HCW’s family. It is notoriously difficult to justify the claim that familial relationships per se generate extra obligations because they are not entered into voluntarily.
